# Requesting conflicts of interest declarations from the European Medicines Agency: 3-year follow-up status

**DOI:** 10.1017/S2045796024000179

**Published:** 2024-03-26

**Authors:** K. Boesen, P. C. Gøtzsche, J. P. A. Ioannidis

**Affiliations:** 1 Meta-Research Innovation Center at Stanford (METRICS), Stanford University, Stanford, CA, USA; 2 Institute for Scientific Freedom, Copenhagen, Denmark; 3 Department of Medicine, Stanford University School of Medicine, Stanford, CA, USA; 4 Department of Epidemiology and Population Health, Stanford University School of Medicine, Stanford, CA, USA

**Keywords:** conflicts of interest, European Medicines Agency, regulatory guidelines, transparency

## Abstract

**Aims:**

We have previously described the European Medicines Agency’s (EMA) and the US Food and Drug Administration’s guidelines, each for a specific psychiatric indication, on how to design pivotal drug trials used in new drug applications. Here, we report on our efforts over 3 years to retrieve conflicts of interest declarations from EMA. We wanted to assess potential internal industry influence judged as the proportion of guideline committee members with industry conflicts of interest.

**Methods:**

We submitted Freedom of Information requests in February 2020 to access EMA’s lists of committee members (and their declared conflicts of interest) involved in drafting the 13 ‘Clinical efficacy and safety’ guidelines available on EMA’s website pertaining to psychiatric indications. In our request, we did not specify the exact EMA committees. Here, we describe the received documents and report the proportion of members with industry interests (i.e. defined as any financial industry relationship). It is a follow-up paper to our first report (http://doi.org/10.1017/S2045796021000147).

**Results:**

After 2 years and 9 months (November 2022), the EMA sent us member lists and corresponding conflicts of interest declarations from the Committee for Medicinal Products for Human use (CHMP) from 2012, 2013 and 2017. These member lists pertained to 3 of the 13 requested guidelines (schizophrenia, depression and autism spectrum disorder). The 10 remaining guidelines were published before 2011 and EMA stated that they needed to require permission from their expert members (with unknown retrieval rate) and foresaw excessive workload and long wait. Therefore, we withdrew our request. The CHMPs from 2012, 2013 and 2017 had from 34 to 36 members; 39%–44% declared any interests and we judged 14%–18% as having industry interests. For the schizophrenia guideline, we identified two members with industry interests to companies who submitted feedback on the guideline. We did not receive declarations from the Central Nervous System (CNS) Working Party, the CHMP appointed expert group responsible for drafting and incorporating feedback into the guidelines.

**Conclusions:**

After almost 3 years, we received information, which only partly addressed our request. We recommend EMA to improve transparency by publishing the author names and their corresponding conflicts of interest declarations directly in the ‘Clinical efficacy and safety’ guidelines and to not remove conflicts of interest declarations after 1 year from their website to reduce the risk of stealth corporate influence during the development of these influential guidelines.

## Background

In 2021, we published a cross-sectional study (Boesen *et al.*, 2021) of the European Medicines Agency (EMA) and the US Food and Drug Administration’s (FDA) regulatory guidance documents on how to design and conduct pivotal clinical trials for psychiatric drug applications. Such regulatory guidelines are likely highly important as the pharmaceutical industry is encouraged to follow them. Despite their potential impact, they have received little academic interest. To our knowledge, only a few studies have assessed their recommendations related to ulcerative colitis (Reinisch *et al.*, 2019) and infectious diseases (Hey *et al.*, 2020), and two papers have assessed general EMA recommendations for psychiatric drug trials, focusing on the choice of comparator (Guaiana and Barbui, 2002) and on how to establish added value for a new drug over existing treatments (Barbui and Bighelli, 2013a). One commentary has highlighted limitations in the EMA guideline on schizophrenia drug trials (Barbui and Bighelli, 2013b). None of these papers focused on potential corporate influence during guideline development.

EMA’s ‘Clinical efficacy and safety’ guidelines fall under the responsibility of the Committee for Medicinal Products for Human use (CHMP) (EMA 2023a), which adopts the guidelines. Since 2010, CHMP appointed domain specific ‘Working Parties’ have drafted and revised the guideline content (EMA, 2023b). Before 2010, the so-called Efficacy Working Party advised EMA on ‘the clinical part of drug development’ across all specialties; there was one expert from each member country (EMA, 2010).

In our first report, we reported the results on three of our project’s four prespecified objectives: (i) guideline development phases, (ii) stakeholder involvement and (iii) trial design recommendations (Boesen *et al.*, 2021). The fourth prespecified objective in our published protocol (Boesen *et al.*, 2020) was to assess the prevalence of declared industry conflicts of interest among those committee members responsible for drafting the guidelines. As neither FDA nor EMA make such information available, we submitted Freedom of Information requests. FDA offered a telephone meeting with an FDA Officer, who informed us that guidelines are drafted in-house and that FDA employees are generally not allowed to have financial conflicts of interest. We found information on FDA’s website that, under some circumstances, employees can also hold financial interests up to $15,000 (FDA, 2017). The EMA responded they were unable to make a timely response due to their relocation from London to Amsterdam, the Netherlands, and because they had two other freedom of information requests from the first author’s then-current affiliation (Cochrane) pending (Boesen *et al.*, 2021). EMA applies a queuing policy (EMA, 2023c) to requests from the same organisations, regardless if the requests are independent of each other, which was the situation in this case.

When we published the results of our study’s first three objectives, 11 months after submitting our freedom of information requests, we had not received any data from EMA. The guidelines influence the design and conduct of pivotal clinical drug trials and shape the evidence bar for new drugs entering the market. Therefore, we anticipated to publish the results of our project’s last objective once receiving the data. This is a 3-year follow-up report describing the conflicts of interest documents subsequently received from the EMA.

## Methods

The study’s methods are described in the protocol (Boesen *et al.*, 2020) and our first report (Boesen *et al.*, 2021). In brief, we aimed to assess four objectives related to regulatory guidance documents published by the EMA and FDA on how to design pivotal clinical drug trials pertaining to psychiatric diagnoses. We included 13 guidelines published by the EMA and 5 by the FDA:

Objective 1: Describing the committee members responsible for drafting the guidelines and their conflicts of interest.

Objective 2: Describing the guideline development phases based on information from the agency websites regarding draft guidelines, public availability, stakeholder involvement, and any restrictions on who could submit feedback throughout the process.

Objective 3: Characterisation of the stakeholders who submitted feedback to draft versions on psychiatric guidelines. We categorised stakeholders according to financial industry relationships (industry; not-industry but with industry related conflicts; independent; unclear).

Objective 4: Describing the guidelines’ design recommendations, focusing on five trial features (duration; exclusion criteria related to psychiatric comorbidities; exclusion criteria related to previous treatment response (so called ‘enriched design’); efficacy outcomes and choice of comparator).

In this follow-up report, we report on Objective 1 based on our subsequent correspondence and documents received from the EMA. One author (KB) assessed the documents.

### Post hoc amendments

We made four adaptations to this follow-up report because our methodology was not described in sufficient detail in our protocol (the first two) and to accommodate the (lack of) data sent by the EMA (the latter two).

#### Categorising conflicts of interest

In our protocol, we stated to report the proportion of committee members with any conflicts of interest (COI). We did not establish criteria to differentiate between ‘industry COI’ (e.g. current employment in the pharmaceutical industry) and ‘other interests’ (e.g. member of a local ethics committee). In this follow-up report, we categorised all financial relations to the pharmaceutical industry as ‘industry COI’ and applied a 36-month threshold, similar to the International Committee of Medical Journal Editors recommendations (ICMJE, 2023). We assumed the conflicts were current if nothing else were stated. One author (KB) judged whether reported disclosures were ‘industry COI’ or ‘other interests’ and ambiguous cases were discussed with the co-authors (PCG, JPAI) for arbitration.

#### Identification of ‘cross-conflicts’

We decided to cross-reference the committee members’ industry conflicts to the stakeholder companies (i.e. those companies that submitted feedback during the guideline development) identified in our first publication (Boesen *et al.*, 2021).

#### Working Party conflicts of interest

EMA did not send us information on the Working Parties involved in the psychiatric guidelines’ development, most importantly the CNS Working Party (EMA, 2023d). To exhaust our options, we sought available online information, including the current member lists and their declared conflicts of interest available from EMA’s database of European Experts, i.e., external members of EMA committees and working groups (EMA, 2023e).

#### Wayback Machine

We used the Wayback Machine website (Archive, 2023), which is a digital library conserving historic and otherwise removed websites, to search for cached and saved versions of EMA’s website for information otherwise not retrievable.

## Results

### Overall results

We received three batches of documents, each pertaining to one guideline; autism spectrum disorder (EMA, 2017), depression (EMA, 2013a) and schizophrenia (EMA, 2012), and we did not receive documents pertaining to 10 guidelines published before 2011 (Table 1). The Wayback Machine archived the EMA website only back to 2018 and we could not retrieve additional information through this channel.

**Table 1.** EMA psychiatric guideline overview

Guideline	Committees	Documents received
Bipolar disorder (2001)	CPMP, EWP	None
Panic disorder (2005)	CHMP, EWP	None
Generalised anxiety disorder (2005)	CHMP, EWP	None
Obsessive-compulsive disorder (2005)	CHMP, EWP	None
Social anxiety (2006)	CHMP, EWP	None
Post-traumatic stress disorder (2008)	CHMP, EWP	None
Alcohol dependence (2010)	CHMP, EWP	None
Attention deficit hyperactivity disorder (2010)	CHMP, EWP	None
Premenstrual dysphoric disorder (2011)	CHMP, EWP, CNS Working Party	None
Insomnia (2011)	CHMP, EWP, CNS Working Party	None
Schizophrenia (2012)	CHMP, CNS Working Party, Biostatistics Working Party, PDCO	18 January 2023
Depression (2013)	CHMP, CNS Working Party, Biostatistics Working Party, PDCO	16 December 2022
Autism spectrum disorder (2017)	CHMP, CNS Working Party	9 November 2022

CHMP = Committee for Medicinal Products in Human use.

CPMP = Committee for Proprietary Medicinal Products (former name of CHMP).

EWP = Efficacy Working Party.

PDCO = Paediatrics Committee.

We received documents related to the CHMP that was involved in drafting all 13 guidelines, but we did not receive information on the relevant CHMP appointed Working Groups; the CNS Working Party (involved in all three assessed guidelines), the Biostatistics Working Party (depression and schizophrenia), the Paediatric Committee (depression) or the Efficacy Working Party that no longer exists (involved in the 10 guidelines we did not receive any information from) (Table 1). Each document package contained (i) a release letter, (ii) a web-link to the corresponding CHMP member list (which is reported in EMA’s annual reports) (EMA, 2023f) and (iii) a collated, unredacted PDF with the CHMP committee’s COI declarations.

### Correspondence with EMA

We submitted our freedom of information request on 10 February 2020 and received the first data batch on 9 November 2022, see detailed timeline in the appendix. Some delay (4 months) may be attributable to the first author’s job change and contact email. Hence, the effective response time was 2 years and 5 months. After receipt of the third batch, EMA prompted us whether we wished to proceed with the remaining request pertaining to guidelines published before 2011, ‘For the upcoming Batch 4 and all batches onwards, the eDOIs to be processed are older than the one processed in batches 1 to 3 (i.e. prior to 2011). These eDOIs were not published on the EMA website. We may have to consult the owners of the eDOIs (the CHMP members) and we, therefore, foresee a difficult consultation period especially as the experts may seized to be CHMP members or even be professionally active any longer’. Disclosure declarations collected before 2011 had seemingly never been released before in any form (e.g. transiently placing them online). We were not able to confirm this on EMA’s website.

This indicated a further substantial long wait on our side, in addition to an excessive workload on EMA’s side. Moreover, we resonated that information might be highly incomplete if members were not possible to reach after so many years (e.g. if they were unavailable, impossible to locate or even deceased) or if they did not provide permission to release that information. On this basis, we decided to withdraw our request for the remaining documents.

### CHMP member lists

The CHMP member lists were from the years 2017 (EMA, 2018), 2013 (EMA, 2014) and 2012 (EMA, 2013b). The number of committee members was 36, 34 and 34, respectively; 39%–44% of the members declared any interest. We categorised 14%–18% of them (5, 6 and 6, in the three guidelines, respectively) as ‘industry COI’ (Table 2; see also the full datafile for details).

**Table 2.** Packages received from EMA

Guideline	CHMP members (*n*)	Members disclosing interests (%)	Industry COIs
Schizophrenia (2012)	34	14 (41%)	6 (18%)
Depression (2013)	34^a^	15 (44%)	6 (18%)
Autism spectrum disorder (2017)	36^b^	14 (39%)	5 (14%)

a 30 overlapping members from 2012; 4 new members.

b 23 overlapping members from 2013; 13 new members.

### Cross-conflicts

Stakeholder comments were not available for 8 of the 13 guidelines (including the depression and autism spectrum disorder guidelines) (Boesen *et al.*, 2021). Thus, we could only assess potential cross-conflicts for the schizophrenia guideline. We categorised two of the six CHMP committee members in 2012, who reported industry COIs, as ‘cross-conflicts’: One CHMP member reported a direct COI (employment less than 3 years ago) to Lundbeck, a company that submitted stakeholder comments on the schizophrenia guideline. The CHMP member had worked for Lundbeck on two schizophrenia drugs. Another CHMP member reported COIs ‘(investigator)’ to two stakeholder companies, Astra Zeneca and Roche, working on various gastrointestinal drugs. A third CHMP member with industry COI (employment less than 3 years ago) to Amgen had worked with them on schizophrenia drugs, but as Amgen had not submitted stakeholder comments, this was not categorised as a ‘cross-conflict’.

### CNS Working Party

We could not retrieve historic CNS Working Party member lists from 2012 (EMA, 2013b), 2013 (EMA, 2014) or 2017 (EMA, 2018); only the Working Party chair and an EMA contact person were reported in the EMA annual reports. The current CNS Working Party (active January 2022–December 2024) consists of eight members; three members reported conflicts, but we did not classify them as ‘industry COI’ (e.g. member of a local ethics committee, husband works as a university professor and employment in the industry 10 years ago).

### Biostatistics working party and paediatric committee

We did not assess the Biostatistics Working Party or the Paediatric Committee as we foresaw similar limitations in retrieving information.

### Efficacy working party

The 10 guidelines not assessed in this follow-up report were all drafted by the Efficacy Working Party (Table 1), which is no longer active and its member lists are no longer available from EMA’s website.

## Discussion

### Main findings

It took patience and perseverance both from us and the EMA to not lose track of our 3-year long inquiry. External factors, e.g. EMA’s relocation and the COVID-19 pandemic, may have contributed to the delay in obtaining the data, and we praise the EMA’s access to document coordinator for being perseverant when our request was ready to be processed. However, a 2-year and 5-month effective delay from inquiry to first received data batch seems disproportionately long. We received unredacted PDFs, which have likely not required other preparation than collating them since the disclosure forms contain the statement, ‘[…] I acknowledge that my information will be stored electronically and published on the EMA website’, and these declarations had been online on EMA’s website before for short periods of time (they are no longer available there).

Current CHMP and other Working Party member lists and COI declarations are publicly available on EMA’s website. However, EMA removes COI declarations 1 year after signature (EMA, 2023e), which is why EMA had to send us these documents rather than simply referring us to their website. It remains unclear why EMA removes COI declarations after 1 year. In EMA’s privacy statement on the handling of expert COI declarations (EMA, 2021), it is noted that EMA keeps COI declarations for 15 years, but neither this statement nor EMA’s Policy 44 (on the handling of expert COIs) (EMA, 2022a) mentions a duration of public online availability. Regrettably, we received disclosure interests only related to the CHMP members but not to the ‘CNS Working Party’ members, i.e. the people who actually drafted the guidelines. The CHMP committee lists are not guideline specific, and it is not possible to infer who were the members most intimately involved in the individual guidelines, and who, potentially, were deemed not eligible due to conflicts of interest. Nevertheless, CHMP members are also very important as they are the ones who choose the CNS Working Party members. It is a limitation to our study that we did not clarify in our freedom of information request that we sought access to both the CHMP and CNS Working Party member declarations. At the time of our request, we did not have sufficient clarity of the EMA infrastructure; thus, we did not consider specifying which exact committees and working groups to ask. Instead, we relied on EMA to exhaustively send us all relevant documentation.

**Figure 1.**
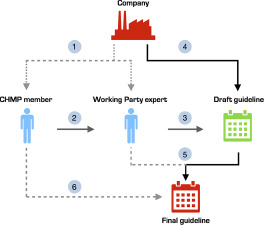
Pathways for corporate influence on EMA regulatory guideline development.

Many disclosed interests were trivial, such as working for a local ethics committee or working in a clinical department where colleagues collaborate with industry. However, we identified some CHMP members with COIs to pharmaceutical companies who submitted stakeholder comments on the draft guidance documents. As noted just above, we cannot ascertain whether potentially conflicted CHMP members were involved in our sample of guidelines. Furthermore, since it is not the CHMP members, but Working Party experts who draft and incorporate feedback into the guidelines, one has to be cautious about whether conflicts of interest of CHMP members may or may not be influential. However, EMA’s current structure leaves room for potential entanglement of industry interests at several levels of the development stage of these highly influential regulatory guidelines (Figure 1).

Companies may influence guideline development directly (solid lines) and indirectly (dotted lines).(1) Pharmaceutical companies may be connected to CHMP committee members and Working Party experts though various conflicts of interest.(2) CHMP committee members appoint Working Party experts.(3) Working Party experts draft EMA guidelines.(4) Pharmaceutical companies can submit stakeholder comments directly on draft guidelines.(5) Working Party experts revise and incorporate feedback into the final guidelines.(6) CHMP committee adopts the final guidelines.

### Findings in context

EMA’s policy on committee members and expert COIs are described in Policy 44 (EMA, 2022a). The policy generally prohibits COIs and requires yearly COI declarations, but there are exceptions depending on the type and ‘level’ of interest as EMA differentiates between ‘direct’ (e.g. employment, consultancy/advisory boards, stocks) and ‘indirect’ (investigator, institutional funding, close family member direct interests) interests. Since 2015, the Agency has published a yearly ‘report on independence’ including a summary of declared COIs. In 2021, 10 (16%) of 63 CHMP members and 781 (20%) of 3868 experts reported ‘direct’ or ‘indirect’ COIs (EMA, 2022b).

In contrast to EMA, our communication with FDA showed that only FDA employees are involved in drafting these influential guideline documents. This alternative path followed by FDA, where guidelines are drafted in-house, may offer in theory pre-emptive protection from COI-related bias. Nevertheless, this can also be contested, since there are multiple levels of interaction between FDA and the industry. Moreover, several FDA officers find lucrative jobs in the industry after leaving FDA (Bien and Prasad, 2016). The influence of the industry on the medical research agenda and standards can take a large number of different forms and may be subtle to decipher (Stamatakis *et al.*, 2013).

The few published studies and articles on regulatory pivotal trial guidance for psychiatry (Barbui and Bighelli, 2013a, 2013b; Guaiana and Barbui, 2002), gastroenterology (Reinisch *et al*., 2019) and infectious diseases (Hey *et al*., 2020) have focused on trial design recommendations only. The potential impact of external and internal industry influence during guideline development (e.g. quantified as the proportion of committee members declaring industry interests) has not been assessed. Therefore, we cannot judge whether corporate influence is more or less frequent in psychiatric guidelines compared to guidelines in other fields.

### Our suggestions

It is standard procedure to disclose COIs in research articles and in clinical guidelines (Lancet, 2019), and we would like to extend our appeal to EMA to apply this principle to their guidance documents. It should be explicitly declared – in the individual guidance documents – which committee members and working group experts that are involved in drafting, revising and approving the final guidelines. Currently, the CHMP member list and the Working Parties’ composition and COIs are published in different places on the EMA website, and it would improve transparency to collect this information in a central registry. We furthermore urge EMA to not remove conflict of interest disclosures from their website after 1 year, after which they are accessible only through freedom of information requests (EMA, 2023e). Historic COI declarations are not kept online for future record even though the documents that were released by old committees may still be active, relevant and influential for research and policy. Also, interestingly, according to our email correspondence, EMA did not disclose COIs before 2011, but we were not able to confirm this from their website. EMA’s initiatives to improve transparency and to avoid corporate influence should be applauded but our case study shows that there is still a need for improvements. We summarise our four specific action points to mitigate stealth corporate influence in Box 1.

## Conclusion

We recommend EMA to improve transparency by reporting explicitly in their clinical efficacy and safety guidance documents who the authors are and their COI declarations. This information should be embedded in the guidance documents themselves rather than being transiently available at some online site. This safeguard seems to be a necessary procedure to reduce the risk of stealth corporate influence at the development stage of these highly influential documents that influence how pivotal industry trials are designed and conducted.

Box 1. Recommendations for action

**Action point 1**. EMA should explicitly disclose names for each CHMP committee member and Working Party expert involved in drafting, revising and approving EMA ‘Clinical efficacy and safety’ guidelines.

**Action point 2**. EMA should explicitly disclose the corresponding conflicts of interest declarations of each involved member and expert involved directly in the guideline development.

**Action point 3**. EMA should not remove conflicts of interest declarations 1 year after signature from their website. These declarations should be kept online for future records to allow retrospective assessment.

**Action point 4**. EMA should curate a digital database of all past and current committees, working groups and their corresponding members. Currently, this information is either scattered across their website, not available, or it is only available through historic documents, such as PDF versions of EMA annual reports.

## Supporting information

Boesen et al. supplementary material Boesen et al. supplementary material

## Data Availability

In our initial freedom of information request to EMA, we stated we would only report summary data and not share data on individual COIs. However, as the received COI declarations have already appeared on EMA’s website, we judged that we could share these documents without compromising confidentiality. We share all documents received by EMA in addition to an Excel sheet summarising the data on the Open Science Framework (https://osf.io/3n284/?view_only=9c13e615057744e7ac4ad1747480b40a).

## Supplementary material

The supplementary material for this article can be found at https://doi.org/10.1017/S2045796024000179.
