# Supplementary material for: Requesting conflicts of interest declarations from the European Medicines Agency: 3-year follow-up status
Source: Epidemiol Psychiatr Sci. 2024 Mar 26;33:e17. doi: 10.1017/S2045796024000179 (PMC11022247; doi:10.1017/S2045796024000179)
Supplement: Boesen et al. supplementary material [file S2045796024000179sup001.pdf]

## Appendix

Boesen K, Gøtzsche PC, Ioannidis JPA. Requesting conflicts of interest declarations from the European Medicines Agency: 3-year follow-up status.

Table 1. Correspondence with EMA

| Date | Content |
|---|---|
| 27 Jan 2020 | Study protocol published on MedRxiv. |
| 10 Feb 2020 | Submission of Freedom of Information (FOI) request to the EMA. |
| 11 Feb 2020 | **EMA**: Automated response acknowledging receipt. |
| 11 Feb 2020 | **EMA**: Asks for clarification of the scope of our submission. |
| 11 Feb 2020 | We respond and confirm the scope of our request. |
| 14 Feb 2020 | **EMA**: Stating that our request is being processed but due to the EMA's relocation there might be delays. Furthermore, two other FOI requests from the first author's "firm" (Cochrane) will be processed before this request. |
| 18 February 2020 | We ask EMA why they state to have two other FOI requests, as we only have knowledge of one other request from the first-author's then-current affiliation (the Nordic Cochrane Centre), which was a request unrelated to the first author. We state it should not affect our request if other Cochrane centres submit requests. |
| 28 February 2020 | **EMA**: Responds, "Indeed this request was submitted by another Cochrane Centre but is placed in the same queue as both the Nordic Cochrane Centre and the other Centre are members of the Cochrane organisation. Kindly note that this approach is in line with the approach applied in the past to the different Cochrane Centres when processing access to documents requests. The Agency applies a queuing system for access to documents requests, in line with the principle of proportionality as set out in its Policy 43, in order to avoid that the core business tasks of the Agency and its performance would be jeopardised by the workload related to activities conducted by the Agency in accordance with Regulation (EC) No 1049/2001." |

| | |
|---|---|
| 29 June 2020 | Follow-up to enquire about status of our request. |
| 30 June 2020 | EMA: Reiterating their response from 14 February 2020 and reiterating that they will contact us then they start processing our request. |
| 30 April 2021 | Study presenting the results of the first 3 objectives of our protocol published in Epidemiology and Psychiatric Sciences. |
| 22 June 2022 | EMA: Contacts Cochrane's Central Executive Team regarding our FOI request. EMA had sent an email to the first author (KB) (unknown what exact date) but due to job change the contact email was no longer active.<br>23 June 2022: Cochrane CET contacts the first author's previous affiliation, Cochrane Denmark.<br>12 Aug 2022: EMA: Follow-up enquiry.<br>25 Aug 2022: The first author is contacted about request.<br>26 Aug 2022: Cochrane Denmark informs EMA about first author's current contact information. |
| 12 Sep 2022 | We follow-up with EMA about enquiry. |
| 20 Oct 2022 | We send EMA a second follow-up email. |
| 20 Oct 2022 | EMA: Response and confirmation that they will now process our request.<br>20 Oct 2022: EMA: Follow-up email that we will receive data on 9 Nov with a possible delay of up to 14 days.<br>20 Oct 2022: We confirm receipt of email. |
| 9 Nov 2022 | EMA: Batch 1 released (autism spectrum disorder). |
| 30 Nov 2022 | EMA: Notification that, "due to current high volume of requests" the deadline for release of Batch 2 is extended to 21 Dec 2022. |
| 16 Dec 2022 | EMA: Batch 2 released (depression guideline). |
| 18 Jan 2023 | EMA: Batch 3 released (schizophrenia guideline). |
| 18 Jan 2023 | EMA: Email from coordinator asking whether we would like to proceed with our request. |

| | |
|---|---|
| 23 Jan 2023 | Response, asking them to clarify continued procedure about receiving one guideline per batch and expected time between each batch. |
| 25 Jan 2023 | **EMA**: Responding that they will continue to send one guideline per batch. Additionally, because the remaining batches pertain to COI declarations from before 2011, these declarations were not published online; EMA therefore has to seek permission from then-CHMP committee members, which could prove laborious. |
| 15 Feb 2023 | **EMA:** Follow-up regarding whether to proceed with request. |
| 15 Feb 2023 | Response, withdrawing the remainder of our request, as it seemed to require excessive efforts to retrieve the remaining COIs in addition to long waiting time on our side to receive the final batches and with uncertain retrieval rate. |
